# Practice effects as a dynamic biomarker of early cognitive change in far-from-onset Huntington’s disease

**DOI:** 10.1093/braincomms/fcag210

**Published:** 2026-06-04

**Authors:** Carla Franch-Marti, Arnau Puig-Davi, Jon Rodriguez-Antiguedad, Jesus Perez-Perez, Gonzalo Olmedo-Saura, Iñigo Ruiz-Barrio, Lidia Bojtos, Laura Perez-Carasol, Adrià Tort-Merino, Javier Pagonabarraga, Jaime Kulisevsky, Saul Martinez-Horta

**Affiliations:** Movement Disorders Unit, Neurology Department, Hospital de la Santa Creu i Sant Pau, 08041 Barcelona, Spain; Research Institute Sant Pau (IR Sant Pau), Parkinson's disease and other Movement Disorders Research Group, 08041 Barcelona, Spain; European Huntington’s Disease Network (EHDN), 89081 Ulm, Germany; Movement Disorders Unit, Neurology Department, Hospital de la Santa Creu i Sant Pau, 08041 Barcelona, Spain; Research Institute Sant Pau (IR Sant Pau), Parkinson's disease and other Movement Disorders Research Group, 08041 Barcelona, Spain; European Huntington’s Disease Network (EHDN), 89081 Ulm, Germany; Centro de Investigación Biomédica en Red-Enfermedades Neurodegenerativas (CIBERNED), 28029 Madrid, Spain; Institute of Neuroscience, Universitat Autònoma de Barcelona (UAB), 08193 Barcelona, Spain; Movement Disorders Unit, Neurology Department, Hospital de la Santa Creu i Sant Pau, 08041 Barcelona, Spain; Research Institute Sant Pau (IR Sant Pau), Parkinson's disease and other Movement Disorders Research Group, 08041 Barcelona, Spain; Centro de Investigación Biomédica en Red-Enfermedades Neurodegenerativas (CIBERNED), 28029 Madrid, Spain; Movement Disorders Unit, Neurology Department, Hospital de la Santa Creu i Sant Pau, 08041 Barcelona, Spain; Research Institute Sant Pau (IR Sant Pau), Parkinson's disease and other Movement Disorders Research Group, 08041 Barcelona, Spain; European Huntington’s Disease Network (EHDN), 89081 Ulm, Germany; Centro de Investigación Biomédica en Red-Enfermedades Neurodegenerativas (CIBERNED), 28029 Madrid, Spain; Movement Disorders Unit, Neurology Department, Hospital de la Santa Creu i Sant Pau, 08041 Barcelona, Spain; Research Institute Sant Pau (IR Sant Pau), Parkinson's disease and other Movement Disorders Research Group, 08041 Barcelona, Spain; European Huntington’s Disease Network (EHDN), 89081 Ulm, Germany; Centro de Investigación Biomédica en Red-Enfermedades Neurodegenerativas (CIBERNED), 28029 Madrid, Spain; Movement Disorders Unit, Neurology Department, Hospital de la Santa Creu i Sant Pau, 08041 Barcelona, Spain; Research Institute Sant Pau (IR Sant Pau), Parkinson's disease and other Movement Disorders Research Group, 08041 Barcelona, Spain; European Huntington’s Disease Network (EHDN), 89081 Ulm, Germany; Centro de Investigación Biomédica en Red-Enfermedades Neurodegenerativas (CIBERNED), 28029 Madrid, Spain; Movement Disorders Unit, Neurology Department, Hospital de la Santa Creu i Sant Pau, 08041 Barcelona, Spain; Research Institute Sant Pau (IR Sant Pau), Parkinson's disease and other Movement Disorders Research Group, 08041 Barcelona, Spain; European Huntington’s Disease Network (EHDN), 89081 Ulm, Germany; Centro de Investigación Biomédica en Red-Enfermedades Neurodegenerativas (CIBERNED), 28029 Madrid, Spain; Movement Disorders Unit, Neurology Department, Hospital de la Santa Creu i Sant Pau, 08041 Barcelona, Spain; Research Institute Sant Pau (IR Sant Pau), Parkinson's disease and other Movement Disorders Research Group, 08041 Barcelona, Spain; European Huntington’s Disease Network (EHDN), 89081 Ulm, Germany; Centro de Investigación Biomédica en Red-Enfermedades Neurodegenerativas (CIBERNED), 28029 Madrid, Spain; Centro de Investigación Biomédica en Red-Enfermedades Neurodegenerativas (CIBERNED), 28029 Madrid, Spain; Alzheimer’s Disease and Other Cognitive Disorders Unit, Hospital Clínic de Barcelona, 08036 Barcelona, Spain; Fundació de Recerca Clínic Barcelona – Institut D’Investigacions Biomèdiques August Pi I Sunyer (IDIBAPS), University of Barcelona, 08036 Barcelona, Spain; Movement Disorders Unit, Neurology Department, Hospital de la Santa Creu i Sant Pau, 08041 Barcelona, Spain; Research Institute Sant Pau (IR Sant Pau), Parkinson's disease and other Movement Disorders Research Group, 08041 Barcelona, Spain; European Huntington’s Disease Network (EHDN), 89081 Ulm, Germany; Centro de Investigación Biomédica en Red-Enfermedades Neurodegenerativas (CIBERNED), 28029 Madrid, Spain; Movement Disorders Unit, Neurology Department, Hospital de la Santa Creu i Sant Pau, 08041 Barcelona, Spain; Research Institute Sant Pau (IR Sant Pau), Parkinson's disease and other Movement Disorders Research Group, 08041 Barcelona, Spain; European Huntington’s Disease Network (EHDN), 89081 Ulm, Germany; Centro de Investigación Biomédica en Red-Enfermedades Neurodegenerativas (CIBERNED), 28029 Madrid, Spain; Movement Disorders Unit, Neurology Department, Hospital de la Santa Creu i Sant Pau, 08041 Barcelona, Spain; Research Institute Sant Pau (IR Sant Pau), Parkinson's disease and other Movement Disorders Research Group, 08041 Barcelona, Spain; European Huntington’s Disease Network (EHDN), 89081 Ulm, Germany; Centro de Investigación Biomédica en Red-Enfermedades Neurodegenerativas (CIBERNED), 28029 Madrid, Spain

**Keywords:** Huntington’s disease, cognition, practice effects, disease monitoring, clinical biomarkers

## Abstract

Although cognitive performance typically appears preserved in far-from-onset Huntingtin Gene Expansion Carriers (HGECs), underlying neuropsychological mechanisms may be subtly altered and remain undetected. Practice effects from repeated task exposure provide a sensitive measure of cognitive adaptability, with early disruptions signalling reduced neural efficiency before standard deficits emerge. This study aimed to examine longitudinal practice effects across annual neuropsychological testing in far-from-onset HGECs and to identify the disease burden threshold where these patterns diverge. Data from 2777 HGECs and 2777 age-matched controls were drawn from the ENROLL-HD cohort across five annual assessments. Longitudinal cognitive trajectories were modelled using linear mixed-effects models, adjusting for demographics. Segmented regression models identified disease burden thresholds marking the onset of practice effect attenuation. Quartile-based stratification assessed the influence of genetic burden. An internet-based Practice Effects Trajectories Calculator (PE-TraC) was developed to model normative longitudinal distributions across tests and visualize individual-level trajectories. HGECs showed reduced practice effects in tasks involving processing speed and executive function, with divergence from controls emerging by Year 2. Segmented regression identified the earliest disease burden breakpoint in Stroop Word Reading Task (249.6) and the latest in the Symbol Digit Modalities Test (291.9). Quartile (Q) analyses showed that participants with the highest genetic burden (Q4) exhibited minimal or absent practice effects, Q3 showed delayed decline, and Q1–Q2 followed similar trajectories to controls. Reduced practice effects constitute a sensitive marker of early cognitive dysfunction in far-to-onset individuals. These findings support the inclusion of dynamic cognitive measures in far-from-onset HGECs trials as potential endpoints.

## Teaser

Franch-Marti *et al*. report that far-from-onset Huntingtin Gene Expansion Carriers show progressive reductions in practice effects, reflecting subtle and dynamic cognitive inefficiencies despite preserved task performance. These changes emerge in a disease burden-dependent manner, supporting practice effects as a sensitive marker for early detection and participant stratification in clinical trials.

## Graphical Abstract

**Graphical Abstract:**
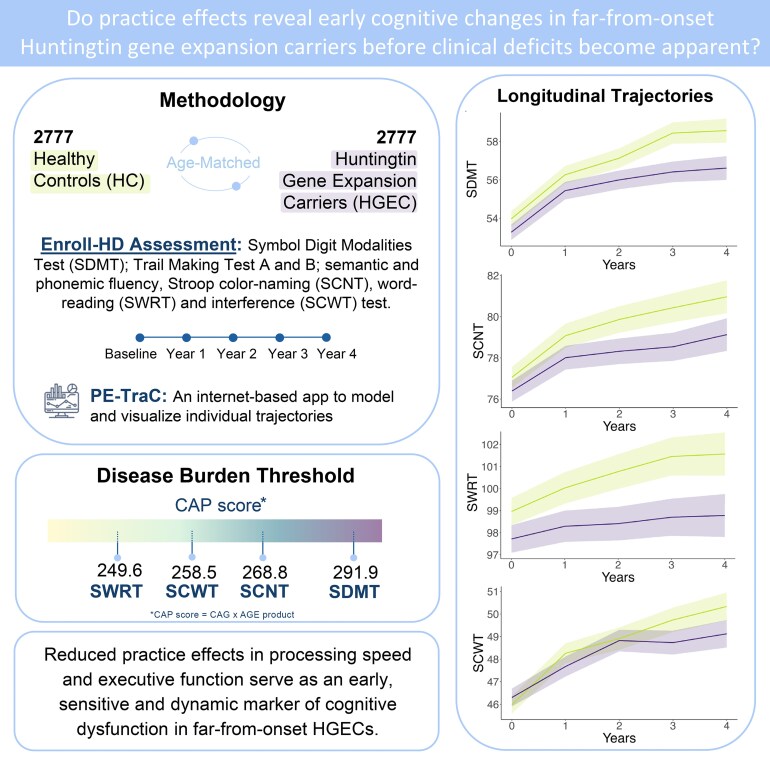

## Introduction

Huntington’s disease (HD) is a progressive, autosomal dominant neurodegenerative disorder caused by an expansion of CAG trinucleotide repeats in the *HTT* gene.^1^ This genetic mutation leads to a progressive neuronal dysfunction and neurodegeneration particularly targeting medium spiny neurons in the striatum, but ultimately affecting the whole brain.^2^ Although the clinical expression of HD varies, individuals carrying a full-penetrance mutation (>39 CAG repeats), referred to as Huntingtin Gene Expansion Carriers (HGECs), are bound to develop symptoms during their lifetime.

Traditionally, HD clinical diagnosis has relied on the emergence of unequivocal motor symptoms.^2^ However, evidence has increasingly shifted this view towards a biological staging framework conceptualizing the disease as a continuum, with neurodegeneration beginning long before motor onset.^3^ In the earliest stages, classified under Huntington’s Disease—Integrated Staging System (HD-ISS) Stages 0 and 1, HGECs may appear clinically asymptomatic, yet subtle biomarkers begin to emerge, signalling the onset of underlying disease processes.^4–6^ Studying these stages is particularly relevant because they represent an optimal window for early therapeutic interventions.

Subtle cognitive changes mostly involving processing speed, have been reported up to 15 years prior to diagnosis.^4,7,8^ Nevertheless, longitudinal studies in large cohorts indicate that throughout HD-ISS 0-1, these changes remain extremely subtle, with no clear differences across multiple cognitive measures compared to non-mutation carriers.^5^ While these findings suggest that there may be an entire stage in which cognitive performance appears preserved, it is also possible that current tests, being largely traditional, may lack the sensitivity to detect subtle progression, whereas more recent or specially designed cognitive tasks have been shown to reveal early longitudinal changes in HGECs.^9,10^ Other factors, such as compensatory mechanisms, may obscure the detection of potential differences.^11^ Accordingly, we may be failing to detect subtle disease-related cognitive changes that are indeed present and hold clinical and research relevance.

In this context, the study of practice effects, defined as the performance improvements observed with repeated exposure to the same task, offers a promising approach to uncovering latent changes in neurocognitive functioning.^12^ These effects reflect the brain’s capacity for learning and adaptation over time. Thus, even when standard cognitive scores do not reveal overt anomalies, examining how individuals benefit, or fail to benefit, from repeated testing may provide a more sensitive measure of early alterations in neural integrity. Previous studies in HD have indeed examined practice effects longitudinally, showing their predictive value for subsequent cognitive decline.^13,14^ However, these studies have primarily focused on individuals already manifesting clinical symptoms, leaving the characterization of practice effects in far-from-onset HGECs largely unexplored. Conceptualizing practice effects not only as potential confounders that can mask gradual cognitive deterioration in early stages but also as early indicators of subtle cognitive dysfunction, could provide a more sensitive framework for detecting preclinical neural changes.

The present study aims to characterize practice effects in a large cohort of far-from-onset HGECs from the Enrol-HD study compared to healthy controls. By analysing longitudinal performance across repeated tasks comprising the cognitive protocol of the Enrol-HD study, we seek to determine whether intraindividual practice effects trajectories differ between groups and whether such differences reflect early subclinical cognitive dysfunction. Furthermore, we aim to estimate the specific cut-off along the disease burden continuum at which these procedural changes diverge from typical patterns.

## Materials and methods

### Study design and participants

This retrospective cohort study was conducted using data from the 6th cut of the Enrol-HD study Periodic Dataset. Enrol-HD study (NCT01574053) is a longitudinal, observational, multinational study aimed to develop a comprehensive repository of prospective and systematically collected clinical research data and biological specimens from individuals with manifest HD, unaffected individuals known to carry the HD mutation or at risk of carrying the HD mutation, and control research participants.^15^

From a global study cohort of 25 550 participants, we focused on far-from-onset HGECs and healthy controls (HC). To avoid effects of reduced penetrance or atypical CAG expansions, inclusion criteria for HGECs were restricted to those with a genetic confirmation of carrying a CAG repeat expansion between 40 and 55. Individuals with repeat lengths greater than 55 were excluded because expansions in this range are more likely to be associated with juvenile-onset or atypical phenotypes, which may follow clinical and neuropsychological trajectories distinct from the adult far-from-onset profile targeted in the present study.^2,3^ Inclusion criteria required a Total Motor Score (TMS) ≤5, a Diagnostic Confidence Score (DCS) <4 and a Total Functional Capacity (TFC) of 13 on the Unified Huntington’s Disease Rating Scale (UHDRS) at baseline.^16^ Additionally, the CAG age product (CAP score) was calculated using the formula [Age × (CAG − 33.66)] to include only participants with a CAP score <350 at baseline, thereby ensuring the selection of individuals corresponding to the HD-ISS Stages 0 and 1.^3,17^ The HC group consisted of non-carriers from HD-affected families, non-biologically related family members, or other community volunteers recruited through HD-related initiatives participating in Enrol-HD. To minimize baseline differences between groups, participants were matched using nearest-neighbour algorithms, pairing each HGEC with the closest HC in terms of age.^18^ Additional matching variables, such as sex and ISCED, were initially considered, but these approaches substantially reduced the available sample size. Therefore, matching based solely on age was retained to preserve statistical power while ensuring comparability between groups. The dataset included five visits, from baseline to four consecutive annual follow-ups. Data beyond Visit 5 were excluded due to substantial attrition, ensuring analytical robustness.

### Standard protocol approvals, registrations and patient consents

The Enrol-HD study is conducted in accordance with the Declaration of Helsinki and the International Conference on Harmonization–Good Clinical Practice (ICH-GCP) guidelines. All participating sites obtained approval from their respective institutional review boards or ethics committees. Written informed consent was obtained from all participants prior to enrolment in compliance with local regulatory requirements. The present work represents a secondary analysis of de-identified data from the Enrol-HD database; therefore, no additional ethical approval or participant consent was required.

### Assessments

The collected demographic data were age, sex, and educational level, assessed using the International Standard Classification of Education (ISCED). Clinical and functional variables included UHDRS-TMS and TFC.

The set of cognitive measures used in the present study comprised all tests from the Enrol-HD protocol, which were specifically selected to target cognitive domains and functions typically affected in HD, such as executive functioning, processing speed, attention, and verbal fluency. Accordingly, we collected data from the Symbol Digit Modalities Test (SDMT), Trail Making Test parts A and B (TMT-A and TMT-B), semantic verbal fluency (animals), phonemic fluency (FAS), Stroop colour naming (SCNT), word-reading (SWRT), and interference (SCWT) test, and the Mini-Mental State Examination (MMSE).

### Statistical analysis

Descriptive statistics were used to summarize demographic and clinical characteristics at each visit, reported as means (standard deviations) for continuous variables and frequencies (percentages) for categorical variables. Cross-sectional between-group differences (HGEC versus HC) were assessed using independent-samples *t*-tests for continuous data and chi-square tests for categorical data, with mean group differences and Cohen’s *d* effect sizes calculated accordingly.

To examine differences in longitudinal cognitive trajectories, linear mixed-effects models (LME) were employed. These models included fixed effects for group (HGECs versus HC), time (years since baseline) and their interaction (group × time) along with age, sex, and ISCED as covariates. A random intercept for each subject was included to account for individual variability; the inclusion of random slopes did not improve model fit or interpretability and was therefore not retained. Model assumptions and covariates’ multicollinearity were checked; model selection was guided by AIC. To account for multiple testing, false discovery rate (FDR) correction was applied using the Benjamini-Hochberg procedure. A detailed description of the handling of missing data and the sensitivity analyses based on multiple imputation is provided in Supplementary Methods 1.

To identify a CAP score cut-off associated with the emergence of reduced practice effects, segmented linear regression analyses were conducted on cognitive tests that exhibited a significant group × time interaction in the previous analyses. For each test, we fitted a linear model including the interaction between time and CAP score. Then, a segmented regression model was applied to estimate the value at which the rate of improvement over time changed significantly. The model also provided 95% confidence intervals for the estimated breakpoint.

Additionally, to better characterize the influence of genetic disease burden on cognitive trajectories, participants were stratified into quartiles based on their baseline CAP score. LME models were then fitted, including quartile subgroup as a fixed effect, its interaction with time, covariates (age, sex, ISCED), and a random intercept per subject. This approach enabled comparison and graphical representation of longitudinal cognitive trajectories across HGEC subgroups with different levels of genetic burden.

All statistical analyses were conducted using R (version 4.4.3). Statistical significance was defined as *P* < 0.05.

### Development of a normative practice effects trajectories calculator (PE-TraC) app

To construct the normative PE-TraC, we modelled one-step change (*Δ* between consecutive visits) in HC across visits 1–5. The four outcome measures were SDMT, SCNT, SWRT, and SCWT.

For each participant and test, consecutive pairs (e.g. V1 and V2, V2 and V3, …) were created, and the following predictors were considered: visit index (as a second-degree polynomial of log(1 + visit)), the one-visit interval, the reference score (baseline at the earlier visit), age, and ISCED. We fitted linear mixed-effects models with a random intercept for subject. These models return the expected stepwise gain conditional on visit index, prior score, and covariates, together with a residual scale for the consecutive step.

Because clinical use often requires comparing non-consecutive visits, we derived the expected change for any pair of visits by chaining the one-step predictions from the control model. Starting from the observed reference score, we iteratively updated the current score with the predicted one-step gain until the comparison visit, summing the gains to obtain the expected multi-step *Δ*. To account for heteroscedasticity across visit gaps, we empirically calibrated the dispersion of residual change as a function of lag, defined as the number of visit intervals between the reference and comparison assessments (e.g. lag = 1 for consecutive visits, lag = 3 for a V1–V4 comparison).

To assess the internal validity of the normative model, HC participants were randomly split at the participant level into training (80%) and test (20%) sets. The normative linear mixed-effects models and lag-specific dispersion parameters were estimated exclusively in the HC training set and subsequently applied, without re-estimation, to the independent HC test set. Validation focused on the distribution of standardized residuals (z-scores), including their centring around zero, dispersion relative to unit variance and empirical coverage within ±1.96 SD. Further technical details and validation results are provided in Supplementary Method 2.

After this validation, the final PE-TraC normative model used in the application was refitted using the full HC sample across Visits 1–5 to maximize precision of the normative estimates. We implemented these procedures in a Shiny application which can be found in https://apuigd.shinyapps.io/PE-TraC/.

## Results

### Sample characteristics

**Figure 1:**
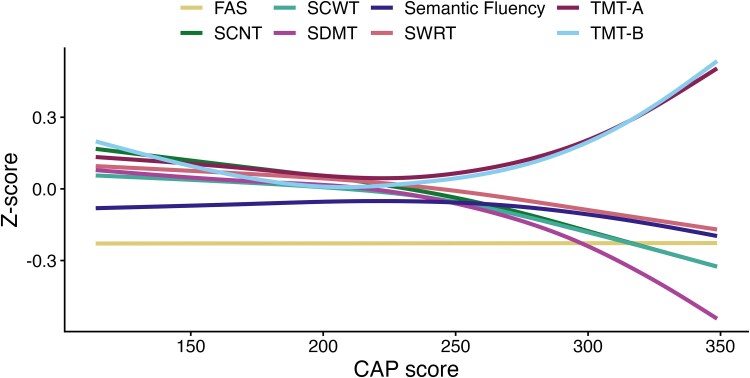
**Estimated trajectories of standardized cognitive performance (*Z*-scores) across CAP scores for each cognitive test.** Smoothed curves are intended to visualize the overall progression for illustrative purposes; all statistical inferences were based on linear models. Analyses were conducted in *N* = 2777 participants.

A total of 2777 age-matched participants were included in each group (HGECs and HC) (Supplementary Fig. 1). Demographic, biological, and clinical data are reported in Table 1 and Supplementary Table 1 provides the number of participants with missing data for each variable. A statistically significant difference in age was found between groups (*P* = 0.003), with a negligible effect size (*d* = −0.102). Sex and educational levels (ISCED) were similarly distributed within groups. Compared to the HC group, HGECs showed slightly higher UHDRS-TMS scores (*P* < 0.001, *d* = 0.261), lower MMSE scores (*P* = 0.022, *d* = −0.100), and lower SWRT (*P* = 0.005, *d* = −0.075). Although these differences reached statistical significance, the absolute group differences were minimal, the effect sizes were small, and all values remained within the clinically normal range, indicating preserved global cognitive and motor functioning. No substantial cross-sectional differences between groups were observed for any of the cognitive measures at either baseline or follow-up visits (see Supplementary Table 2). Figure 1 illustrates the estimated trajectories of standardized cognitive performance (*Z*-scores) across the CAP score continuum for each cognitive test, depicting how cognitive functioning changes as disease burden increases.

**Table 1:** Clinical and sociodemographic characteristics of the sample at baseline

	HGECs *N* = 2777	HC *N* = 2777	*P*-value	Cohen’s *d*
Age	33 (8)	34 (8)	**<0**.**001**	−0.102
Sex			0.336	
Female	1702 (61%)	1667 (60%)		
Male	1075 (39%)	1110 (40%)		
ISCED			**0**.**004**	
1	18 (0.7%)	46 (1.7%)		
2	188 (6.8%)	225 (8.1%)		
3	721 (26%)	709 (26%)		
4	630 (23%)	588 (21%)		
5	1107 (40%)	1099 (40%)		
6	104 (3.8%)	96 (3.5%)		
CAG	42.27 (2.05)	NA		
CAP score	273 (51)	NA		
TFC	13 (0)	13 (0)		
UHDRS-TMS	1.03 (1.48)	0·68 (1.20)	**<0**.**001**	0.261
MMSE	28.96 (1.36)	29·09 (1.32)	**0**.**001**	−0.100
SDMT	54 (10)	54 (11)	0.076	−0.048
SCNT	77 (13)	77 (13)	0.106	−0.043
SWRT	98 (17)	99 (16)	**0**.**005**	−0.075
SCWT	47 (10)	46 (10)	0.093	0.046
Semantic fluency	22.4 (5.5)	22.6 (5.6)	0.103	−0.044
FAS	41 (12)	41 (12)	0.291	−0.031
TMT-A	25 (11)	24 (11)	0.161	0.041
TMT-B	52 (25)	51 (25)	0.384	0.026

Data are expressed as mean (SD) or *n* (%). HGECs, Huntingtin Gene Expansion Carriers; HC, healthy controls; ISCED, International Standard Classification of Education; CAP score, CAG age product; TFC, total functional capacity; UHDRS-TMS, Unified Huntington’s Disease Rating Scale—Total Motor Score; MMSE, Mini-mental State Examination; SDMT, Symbol Digit Modalities Test; SCNT, Stroop Colour Naming Task; SWRT, Stroop Word Reading Task; SCWT, Stroop Colour-Word Task; FAS, phonemic fluency; TMT, trail making test. Statistically significant results (*P* < 0.05) are highlighted in bold.

### Linear mixed-effects models

**Figure 2:**
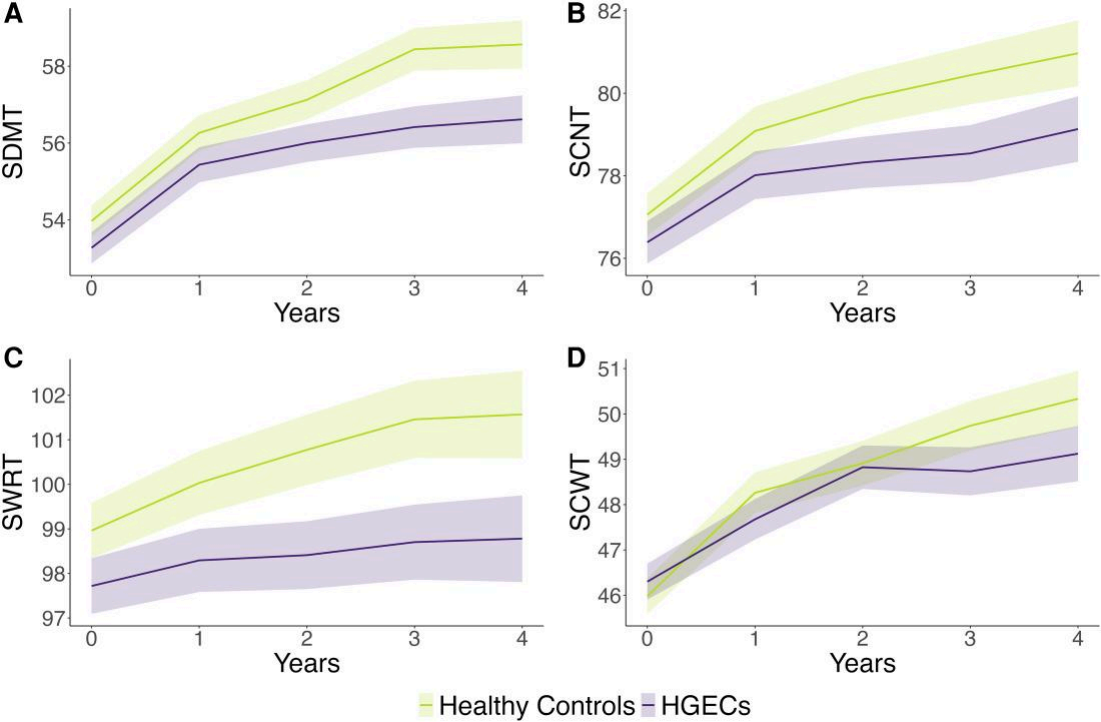
**Longitudinal trajectories by cognitive test according to the linear mixed-effects models.** Panels **A–D** display the longitudinal trajectories for the SDMT, SCNT, SWRT, and SCWT, respectively. The *Y*-axis in all panels represents the raw scores of the respective cognitive tests. Analyses were conducted in *N* = 5554 participants.

Longitudinal analysis using LME models revealed significant group-by-time interactions in the SDMT and the Stroop tests (Fig. 2), indicating divergent practice effects trajectories between HGECs and HC (Supplementary Table 3).

Specifically, for the SDMT, HC continued to improve by approximately 4.5 points from baseline to Year 3, whereas HGECs showed a significantly smaller gain, with a between-group difference of −1.33 points (*P* < 0.001) at Year 3 and −1.25 points (*P* = 0.002) at Year 4. For the Stroop tasks, differences emerged earlier and were more pronounced over time. In SCNT, HGECs gained 0.88 fewer points than HC at Year 2 (*P* = 0.021), a gap that widened to −1.23 points at Year 3 (*P* = 0.005) and persisted at Year 4 (*β* = −1.17, *P* = 0.022). In SWRT, the divergence was evident as early as Year 2 (*β* = −1.12, *P* = 0.017) and continued to increase, reaching −1.54 points at Year 4 (*P* = 0.014). For SCWT, the interaction became significant at Year 3 (*β* = −1.32, *P* < 0.001) and further strengthened by Year 4 (*β* = −1.53, *P* < 0.001).

In contrast, no such interactions were observed for other tasks such as FAS, Semantic Fluency, TMT-A, and TMT-B, where both groups showed comparable gains over time.

These findings were confirmed in sensitivity analyses using multiple imputation of missing data, which yielded similar results to the primary LME models (Supplementary Table 4), indicating that missing data had minimal impact on the observed group-by-time interactions.

### Segmented regression analysis

Segmented regression analysis revealed a significant breakpoint in the relationship between CAP score and each cognitive measure. Specifically, the results suggested a temporal sequence in the reduction of practice effects across cognitive domains. The earliest attenuation was observed in the SWRT, with a breakpoint estimated at a CAP score of 249.6 (95% CI: 210.3–288.8). This was followed closely by the SCWT at 258.5 (CI: 228.9–288.1) and the SCNT at 268.8 (CI: 237.4–300.3). Finally, the SDMT showed a later inflection point at 291.9 (CI: 272.7–311.1). The complete model estimates are presented in Supplementary Tables 5 and 6.

### Linear mixed-effects analysis stratified by CAP score quartiles

LME models examining the influence of baseline genetic burden revealed distinct cognitive trajectories over time (Supplementary Table 7). Participants were stratified into quartiles of CAP score: Q1 (114.12–237.82), Q2 (237.83–280.20), Q3 (280.21–316.92), and Q4 (316.93–348.70).

Taking Q1 as the reference, individuals in Q4 (representing the highest genetic burden) showed significantly attenuated or absent practice effects from the earliest follow-ups across all tasks. Participants in Q3 initially showed improvements comparable to Q1, but these gains progressively declined from Year 3 (*β*_SDMT_ = −2.05, *P* = 0.001; *β*_SCNT_ = −1.74, *P* = 0.039; *β*_SWRT_ = −3.30, *P* = 0.001; *β*_SCWT_ = 1.80, *P* = 0.005). Despite this reduction, Q3 participants generally retained 1–3 points above baseline across most tasks. In contrast, Q2 individuals maintained practice effects trajectories similar to Q1, with non-significant interaction terms across follow-up years, reflecting preserved practice effects and cumulative gains of 3–4 points above baseline throughout follow-up.

**Figure 3:**
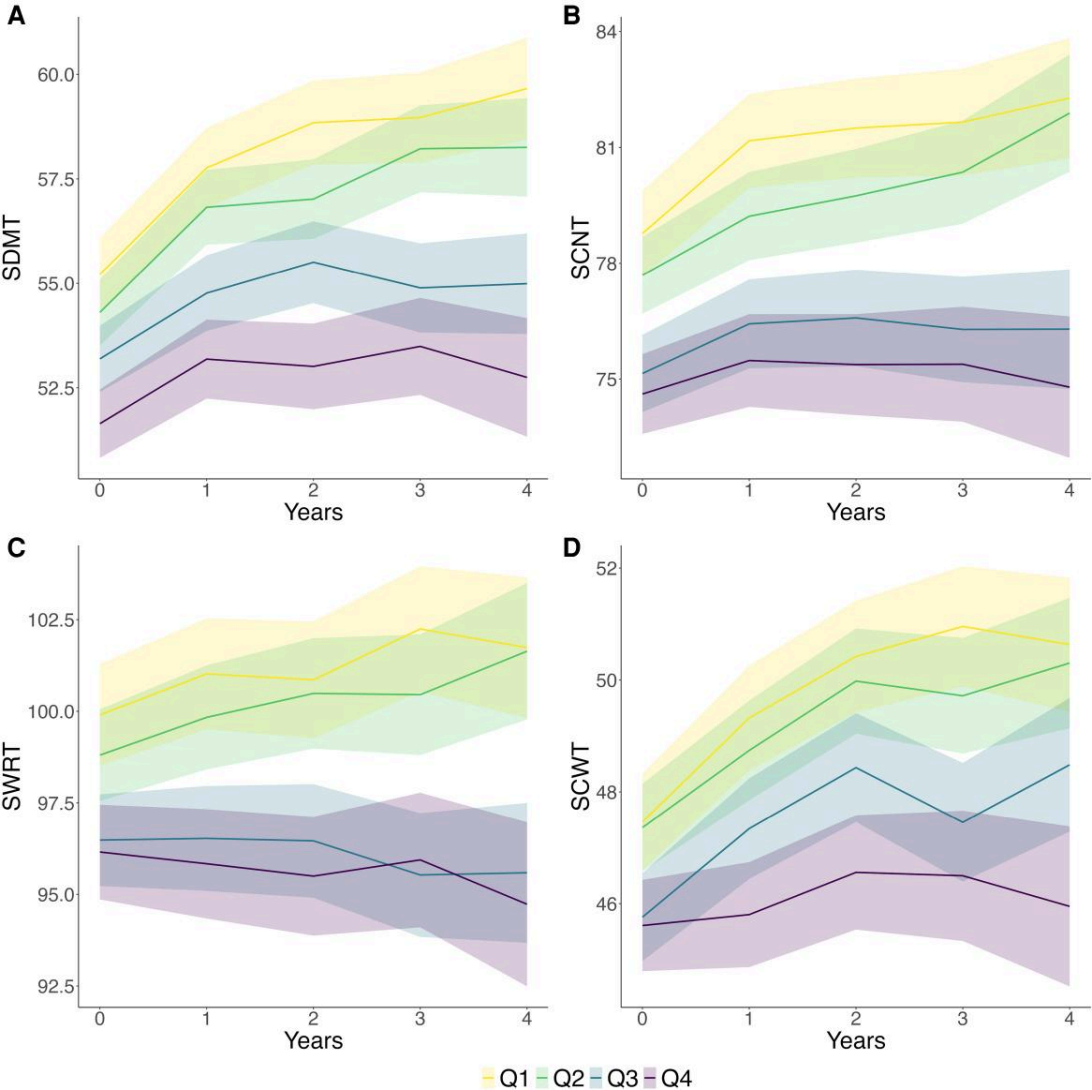
**Longitudinal trajectories by CAP-score quartiles of the different cognitive tests according to the linear mixed-effects models.** Panels **A–D** display the longitudinal trajectories by CAP-score quartiles for the SDMT, SCNT, SWRT, and SCWT, respectively. The *Y*-axis in all panels represents the raw scores of the respective cognitive tests. Analyses were conducted in *N* = 5554 participants.

Graphical representations of these trajectories are shown in Fig. 3.

### PE-TraC internet-based app

The PE-TraC tool enables the input of individual patient data, including age, educational level (ISCED), CAG, the cognitive test of interest, the selected reference and comparison visits, and their corresponding scores. It computes the observed change between visits and compares it to the expected change derived from the built normative model.

Outputs include the expected and observed deltas, a standardized *Z*-score quantifying the deviation of the observed change from the normative expectation, the number of matched controls available for the selected comparison, and a trajectory plot visualizing the patient’s cognitive change in the context of the normative distribution.

Internal validation of the normative model was performed in an independent HC test set comprising 348 participants after model training in 1391 HC participants. Across the four cognitive tests, standardized residuals were approximately centred around zero (mean *z* range: −0.14 to −0.05), with standard deviations ranging from 1.12 to 1.28. The proportion of observations within ±1.96 SD ranged from 88.2% to 92.7%, supporting reasonable out-of-sample calibration of the normative model. Full validation metrics are reported in Supplementary Tables 8 and 9.

## Discussion

In this large cohort study, cognitively unimpaired HGECs exhibited distinct practice-effects trajectories compared to HC. While cross-sectional cognitive assessments revealed no significant differences, longitudinal analyses uncovered an attenuation of practice effects in far-from-onset HGECs, especially in processing speed and executive tasks. These findings extend previous observations in manifest HD^13,14^ by demonstrating that alterations in practice effects emerge much earlier, prior to measurable cognitive decline, suggesting that such dynamics can serve as a marker in HD-ISS 0-1 clinical trials.

From a clinical perspective, the SDMT and Stroop subtests emerged as the most sensitive indicators of these trajectory differences. These measures rely on fronto-subcortical pathways vulnerable in HD^19^ and are also key components of the composite UHDRS (cUHDRS), a widely validated index used to monitor disease progression and as a primary endpoint in clinical trials.^20^

Previous studies have identified a range of longitudinal cognitive markers sensitive to decline in early stages of HD, particularly within executive functions, but also affecting emotional recognition and visuospatial domains.^5,21,22^ While these markers have significantly advanced early detection, they predominantly capture absolute performance levels and may not fully reflect the dynamic properties of cognitive functioning. In this context, practice effects offer a complementary perspective by indexing experience-dependent adaptive processes, revealing how individuals optimize performance through repeated task exposure. As such, they reflect not only task proficiency but also the integrity of neural mechanisms supporting skill acquisition, automatization, and the progressive optimization of cognitive efficiency over time. Within this framework, the selective attenuation of practice effects observed in far-from-onset HGECs suggests an early disruption of these adaptive processes, revealing subtle alterations in system-level functioning that remain undetected by standard cognitive metrics. This dissociation underscores the conceptual distinction between preserved cognitive capacity and impaired adaptability, with important implications for early disease detection, mechanistic understanding, and biomarker development.

Notably, the attenuation of practice effects followed a progressive and domain-specific temporal sequence, closely aligned with genetic disease burden. In line with the well-established relationship between higher genetic load and faster disease progression in HD,^7,22^ stratification by CAP score quartiles revealed that individuals with the highest genetic burden exhibited an earlier and more pronounced reduction in practice effects, whereas those with intermediate CAP scores showed a more gradual decline. These findings position practice effects not merely as epiphenomena of cognitive dysfunction, but as dynamic markers reflecting early alterations in neural plasticity and network efficiency along the biological continuum of HD.

From a mechanistic perspective, neurobiological evidence provides a plausible mechanistic substrate for these findings. Structural imaging studies have consistently identified striatal atrophy, particularly within the putamen and caudate nucleus, even in individuals far from clinical diagnosis.^23^ These regions are critical hubs in cortico-striatal loops that support procedural learning, attentional control, and processing speed, domains that are critically engaged in the tasks where diminished practice effects were observed. In turn, these circuits heavily rely on dopaminergic transmission, whose disruption has been well-documented in HD^24,25^ and may therefore impair the adaptive neural responses required to benefit from repeated cognitive testing.

Beyond fronto-striatal dysfunction, practice effects critically depend on hippocampal integrity to support rapid learning, as well as the integration and consolidation of contextual and perceptual information during initial encoding.^26^ Through these mechanisms, the hippocampus enables individuals to extract regularities, detect stimulus relationships, and efficiently deploy prior experience to optimize subsequent performance.^27^ Consistent with this framework, patients with focal hippocampal damage show markedly attenuated practice effects, highlighting the necessity of intact hippocampal circuitry for normal learning-related gains.^28^ In HD, converging evidence indicates early and selective vulnerability of hippocampal subregions closely associated with the perforant pathway and memory processing.^29^ Even in HD-ISS 1, when global hippocampal volume may remain relatively preserved, subtle microstructural and functional alterations have been reported, potentially affecting network-level learning efficiency before overt neuronal loss becomes evident.^30,31^ Within this context, accelerated forgetting between visits represents one possible explanation; however, although this phenomenon is well established in Alzheimer’s disease, it remains insufficiently characterized in HD and should therefore be interpreted cautiously. Alternatively, and perhaps more consistently with the known pathophysiology of HD, attenuated practice effects may reflect early impairments in procedural and frontostriatal-dependent learning mechanisms rather than true acceleration of memory decay. Future studies directly comparing retest effects across shorter versus longer inter-assessment intervals would be instrumental in disentangling reduced acquisition, impaired consolidation, and accelerated forgetting as potential contributors to these longitudinal patterns.

The CAP score itself has been linked to greater cortical and subcortical atrophy, increased mutant huntingtin (mHTT) expression, and elevated neurofilament light chain (NfL) levels in fluid biomarkers.^5,6,32^ Furthermore, higher CAP scores have been associated with early disruptions in brain network architecture, including altered hub connectivity and reduced integration across large-scale neural systems.^33^ Such changes suggest a progressive decline in the brain’s capacity to efficiently process information, even during the preclinical phase. The gradual attenuation of practice effects observed across increasing CAP score quartiles may reflect these progressive neuropathological changes compromising cognitive reconfiguration over time.

Importantly, this attenuation in practice-related gains occurred despite preserved performance on traditional cross-sectional measures, which typically remain within the normal range until later disease stages. In this context, the ability to capture subtle changes in cognitive dynamics during HD-ISS Stages 0 and 1 becomes particularly relevant, as practice effects may provide a sensitive marker of early cognitive vulnerability, preceding overt cognitive decline. By contrast, in other stages like HD-ISS 2, reduced or absent practice effects may possibly reflect a genuine decline in cognitive performance rather than a true absence of learning, highlighting the need for careful interpretation of this phenomenon across disease stages.

Growing evidence indicates that far-from-onset HGECs maintain cognitive performance via compensatory strategies, such as increased recruitment of non-traditional cortical regions or greater executive resource allocation.^34,35^ Functional neuroimaging studies have shown reduced connectivity within frontostriatal and frontoparietal networks during executive tasks in these stages,^34^ as well as broader disruptions in large-scale networks, including the default mode and salience networks, commonly implicated in compensatory mechanisms and early network failure.^36^ In parallel, PET studies have demonstrated early hypometabolism in associative cortical areas^37^ and in the striatum, even before clinical onset, further indicating metabolic compromise in preclinical stages.^38^ Together, these findings may help explain why such compensatory strategies, while effective in preserving baseline performance, may come at the expense of reduced cognitive plasticity. That is, although task accuracy is maintained, the capacity to benefit from repeated testing is reduced. This dissociation reinforces the value of dynamic testing paradigms for capturing subtle functional decline in preclinical stages.

To address this gap, we developed PE-TraC, a normative framework that provides dynamic, individualized estimates of expected retest-related gains over time. By visualizing patient-specific trajectories against normative reference models derived from HC, the tool enables the detection of subtle deviations that are not captured by static scores alone. Its outputs include expected and observed change, the corresponding standardized *z*-score, and a trajectory plot contextualizing the individual’s cognitive change within the normative range. This feature has significant implications for clinical monitoring and trial design, as deviations in practice effects could represent a sensitive marker of disease progression. Furthermore, PE-TraC may support more refined stratification of individuals across HD-ISS stages, particularly within the preclinical range (Stages 0–1), and thereby enhancing early-stage characterization and participant selection in preclinical studies.^3^

Despite the strengths of our study, several limitations should be noted. First, the multicentric nature of the Enroll-HD database may introduce some variability in data collection procedures, such as differences in test administration order and inter-rater reliability, which could affect the consistency of cognitive assessments across sites. In addition, baseline was defined as each participant’s first visit following enrolment in Enroll-HD. However, it cannot be guaranteed that this visit corresponds to the participant’s first-ever exposure to the cognitive tests included in this study, as prior assessments outside of Enroll-HD cannot be fully ruled out. Such prior exposure could influence performance through practice effects, introducing additional uncontrolled variability. The absence of concomitant neurobiological biomarkers limits the ability to directly relate cognitive changes to underlying neuropathological processes. Moreover, interindividual variability in disease progression, potentially influenced by factors such as lifestyle, comorbidities or genetic modifiers, may complicate the interpretation of the observed patterns of practice effects.

Future research should combine broader cognitive assessments with multimodal neuroimaging and biomarkers to better characterize early dysfunctions in HGECs and their underlying correlates. Furthermore, validating the PE-TraC tool in independent, prospectively collected cohorts will be crucial to confirm its robustness and generalizability.

In conclusion, this study demonstrates that reduced practice effects in processing speed and executive function serve as an early, sensitive, and dynamic marker of cognitive disruption in far-from-onset HGECs. By incorporating these findings into a practical internet-based app, we provide a novel tool that not only enables disease progress monitoring but also opens the possibility of establishing practice effects as robust clinical trial endpoints in the earliest stages of the disease.

## Supplementary Material

fcag210_Supplementary_Data

## Data Availability

The dataset used in this study was obtained from the Enroll-HD global clinical research platform (https://www.enroll-hd.org/). Enroll-HD provides de-identified clinical data to qualified researchers through periodic data sets (PDS), released every 1–2 years after extensive quality control and anonymization procedures. Access to more detailed, non-aggregated data can be requested through a specified dataset (SPS) application, subject to approval by the Scientific Review Committee of the CHDI Foundation. More information on data access procedures is available on the Enroll-HD website. The R scripts used for data preprocessing and statistical analyses are publicly available on GitHub at: https://github.com/cfranch/R-code/.
