# Supplementary material for: Practice effects as a dynamic biomarker of early cognitive change in far-from-onset Huntington’s disease
Source: Brain Commun. 2026 Jun 4;8(3):fcag210. doi: 10.1093/braincomms/fcag210 (PMC13284418; doi:10.1093/braincomms/fcag210)
Supplement: fcag210_Supplementary_Data [file fcag210_supplementary_data.pdf]

**Supplementary Appendix**

Supplementary Methods 2
1. Handling of missing data 2
2. PE-TraC development 2

Supplementary Figures 4
Figure 1. Study flowchart 4

Supplementary Tables 5
Table 1. Missing data at each visit 5
Table 2. Cognitive and sociodemographic characteristics at each visit 6
Table 3. LME model coefficients 8
Table 4. Comparison of original and MICE-imputed linear mixed-effects model estimates for cognitive outcomes 10
Table 5. Segmented regression analysis 11
Table 6. Estimated breakpoints 12
Table 7. LME analysis stratified by CAP score quartiles 13
Table 8. Internal validation of the normative PE-TraC model in the held-out HC test set 14
Table 9. Internal validation of the normative PE-TraC model by visit lag in the held-out HC test set 15

# Supplementary Methods

## 1. Handling of missing data

To assess the robustness of our findings, sensitivity analysis was conducted using multiple imputation by chained equations (MICE) package in R.

The imputation included all cognitive outcomes and relevant covariates Predictive mean matching (PMM) was applied to all continuous variables. Ten imputed datasets were generated over 20 iterations. A random seed was fixed to ensure reproducibility. Following imputation, the same LME models used in the primary analysis were re-fitted separately to each imputed dataset and results were pooled according to Rubin's rules. Effects estimates and significance levels were then compared with those obtained from the original, non-imputed dataset.

As reported in Supplementary Table 4, the direction and magnitude of all interaction effects were highly consistent between the original and MICE-imputed models. No notable discrepancies in significance were observed. These findings indicate that missing data had minimal impact on our longitudinal cognitive results, supporting the robustness of the main analyses.

## 2. PE-TraC development

### a) Data preprocessing

For each analysis unit (subject×test×pair), rows with missing scores at either visit were excluded for that pair only. Age and ISCED were used as covariates for the modeling steps described below.

### b) Consecutive step model in controls

For each test, we constructed a stepwise dataset of consecutive transitions within subjects, recording baseline_prev (score at the earlier visit), delta (one-step change), start_seq, interval_y=1, age, and ISCED. We fit:

$$\Delta = \beta^0 + \beta^1 \cdot poly(log(1 + start\_seq), 2) + \beta^2 \cdot interval\_y + \beta^3 \cdot baseline\_prev + \beta^4 \cdot Age + \beta^5 \cdot ISCED + u(subjid) + \varepsilon$$

using restricted maximum likelihood. The fitted object provides (i) a prediction function for one-step gains and (ii) the residual σ for the consecutive step.

### c) Chaining procedure

For any pair of visits (Reference (Ref) and Comparison (Comp)), we initialized the current score at the observed Ref score and iterated stepwise from Ref to Comp−1, each time predicting the one-step gain from the control model given the current score, visit index, age, and ISCED, updating the current score and accumulating the expected Δ. This yields the expected multi-step change between Ref and Comp. This chaining ensures that expected gains reflect cumulative practice effects rather than a single-step extrapolation.

### d) Lag-specific dispersion

Using Healthy Controls only, we enumerated all valid visit pairs within each subject and calculated the residuals as the difference between the observed and the chained expected change

($\Delta obs - \Delta exp$). These residuals were then grouped by lag, defined as the number of visit intervals between the reference and comparison assessments (e.g., lag = 1 for consecutive visits, lag = 3 for a V1–V4 comparison). Because the variability of these residuals naturally increases with longer intervals, we empirically estimated a separate dispersion term for each lag. For lags with sufficient data ($n \geq 50$) and a finite Median Absolute Deviation (MAD) (a robust measure of spread less influenced by outliers) the dispersion was estimated as 1.4826 × MAD, corresponding to an equivalent standard deviation under normality. When the sample size for a given lag was small or the MAD was undefined, the conventional standard deviation was used instead. If no empirical estimate was available for a particular lag, the effective standard deviation $\sigma\_eff(lag)$ was approximated by scaling the consecutive-step residual $\sigma$ by $\sqrt{lag}$, assuming variance accumulates proportionally with time separation.

**e) Internal validation of the normative model**

To assess internal validity, Healthy Controls were randomly split at the participant level into training (80%) and test (20%) sets. The consecutive-step mixed-effects models and lag-specific dispersion estimates were fitted exclusively in the HC training set and then applied, without re-estimation, to the independent HC test set.

In the held-out HC sample, we generated all valid visit pairs for each subject and test, computed the observed change ($\Delta obs$), the chained expected change ($\Delta exp$), and the lag-specific effective standard deviation, and then derived a standardized deviation score as:

$$z = (\Delta obs - \Delta exp) / \sigma eff$$

Validation focused on whether these standardized residuals were approximately centered around zero, showed dispersion close to unit variance, and achieved empirical coverage near the expected range within ±1.96 SD. We additionally summarized performance by visit lag to examine whether calibration remained stable across different inter-visit intervals.

## Supplementary Figures

**Supplementary Figure 1. Study flowchart**

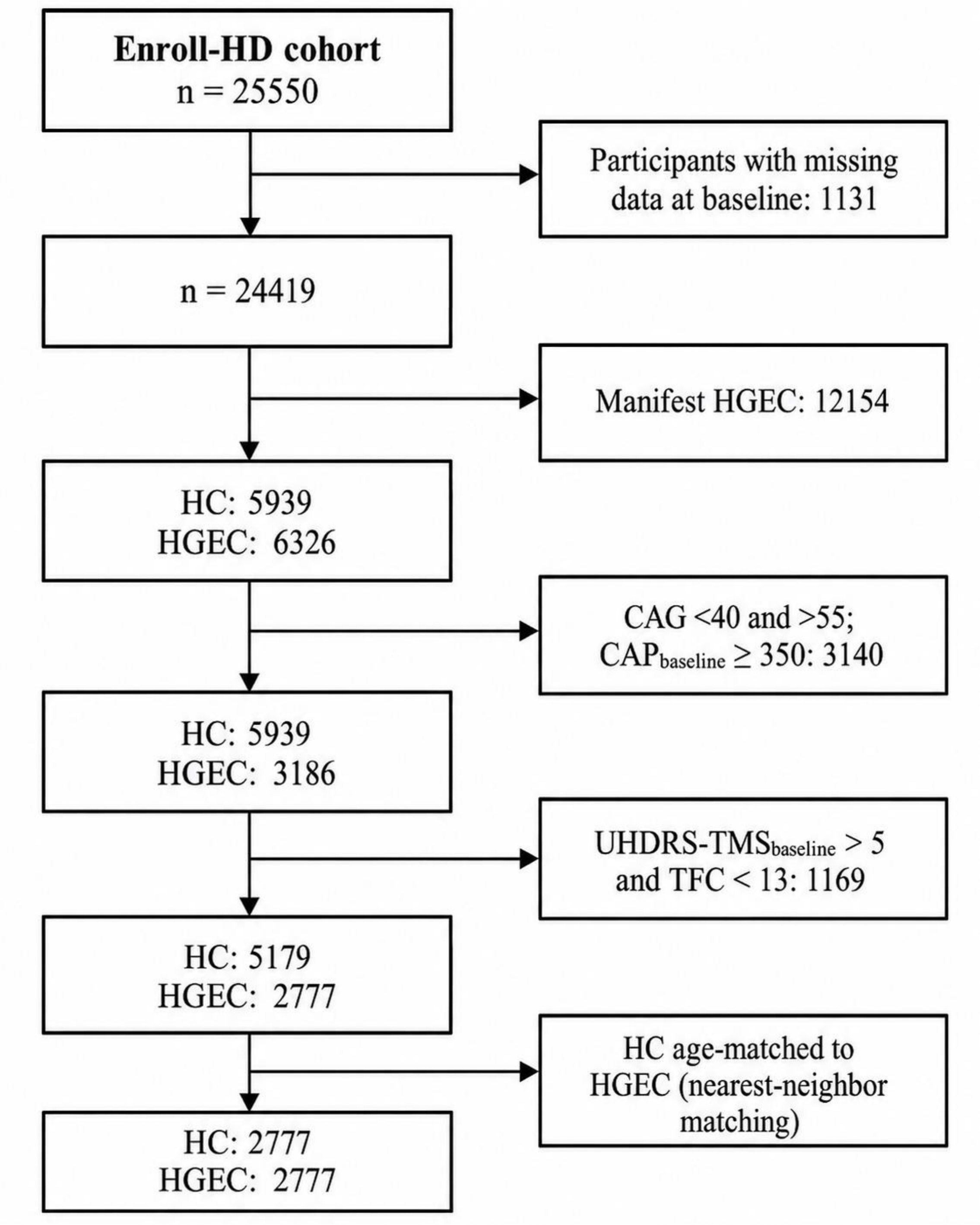


Flow diagram illustrating participant selection from the ENROLL-HD dataset, inclusion and exclusion criteria and the final sample used for the analyses. *Abbreviations*: HGECs, Huntingtin Gene Expansion Carriers; HC, Healthy Controls; CAP baseline, CAG age product at baseline; UHDRS-TMS, Unified Huntington's Disease Rating Scale – Total Motor Score; TFC, Total Functional Capacity.

# Supplementary Tables

**Supplementary Table 1. Missing data at each visit**

| | V1 | | V2 | | V3 | | V4 | | V5 | |
|---|---|---|---|---|---|---|---|---|---|---|
| | **HGECs** N = 2777 | **HC** N = 2777 | **HGECs** N = 1600 | **HC** N = 1534 | **HGECs** N = 1208 | **HC** N = 1090 | **HGECs** N = 858 | **HC** N = 810 | **HGECs** N = 551 | **HC** N = 554 |
| **Age** | 0 | 0 | 0 | 0 | 0 | 0 | 0 | 0 | 0 | 0 |
| **Sex** | 0 | 0 | 0 | 0 | 0 | 0 | 0 | 0 | 0 | 0 |
| **ISCED** | 9 | 14 | 3 | 8 | 3 | 3 | 1 | 1 | 0 | 1 |
| **CAG** | 0 | - | 0 | - | 0 | - | 0 | - | 0 | - |
| **CAP score** | 0 | - | 0 | - | 0 | - | 0 | - | 0 | - |
| **TFC** | 0 | 0 | 0 | 0 | 0 | 0 | 0 | 0 | 0 | 0 |
| **UHDRS-TMS** | 0 | 0 | 0 | 0 | 0 | 0 | 0 | 0 | 0 | 0 |
| **MMSE** | 722 | 665 | 383 | 330 | 297 | 248 | 201 | 178 | 134 | 117 |
| **SDMT*** | 3 | 6 | 3 | 3 | 4 | 3 | 1 | 3 | 3 | 3 |
| **SCNT*** | 8 | 13 | 6 | 5 | 5 | 3 | 2 | 5 | 2 | 1 |
| **SWRT*** | 5 | 10 | 3 | 3 | 3 | 2 | 1 | 3 | 0 | 0 |
| **SCWT** | 151 | 119 | 73 | 72 | 61 | 51 | 51 | 46 | 25 | 38 |
| **Semantic Fluency*** | 10 | 7 | 0 | 3 | 4 | 4 | 1 | 1 | 0 | 0 |
| **FAS** | 470 | 473 | 223 | 241 | 179 | 172 | 121 | 132 | 77 | 98 |
| **TMT-A** | 468 | 442 | 213 | 222 | 162 | 144 | 113 | 109 | 68 | 86 |
| **TMT-B** | 468 | 442 | 213 | 222 | 162 | 144 | 113 | 109 | 68 | 86 |

*Note*: * These tests are part of the Enroll-HD Core Cognitive Assessment. The remaining tests belong to the Extended Cognitive Assessment, which includes additional, non-mandatory measures.
*Abbreviations*: HGECs, Huntingtin Gene Expansion Carriers; HC, Healthy Controls; ISCED, International Standard Classification of Education; CAP score, CAG age product; TFC, Total Functional Capacity; UHDRS-TMS, Unified Huntington's Disease Rating Scale – Total Motor Score; MMSE, Mini-mental State Examination; SDMT, Symbol Digit Modalities Test; SCNT, Stroop Color Naming Task; SWRT, Stroop Word Reading Task; SCWT, Stroop Color-Word Task; FAS, phonemic fluency; TMT, Trail Making Test.

**Supplementary Table 2. Cognitive and sociodemographic characteristics at each visit**

| | V2 | | | | V3 | | | |
|---|---|---|---|---|---|---|---|---|
| | **HGECs** N = 1600 | **HC** N = 1534 | **p-value** | **Cohen's d** | **HGECs** N = 1208 | **HC** N = 1090 | **p-value** | **Cohen's d** |
| **Age** | 34 (9) | 36 (8) | **<0.001** | 0.157 | 35 (8) | 37 (8) | **<0.001** | 0.225 |
| **Sex** | | | 0.916 | | | | 0.695 | |
| female | 989 (62%) | 951 (62%) | | | 744 (62%) | 680 (62%) | | |
| male | 611 (38%) | 583 (38%) | | | 464 (38%) | 410 (38%) | | |
| **ISCED** | | | 0.096 | | | | **<0.001** | |
| 1 | 12 (0.8%) | 28 (1.8%) | | | 7 (0.6%) | 23 (2.1%) | | |
| 2 | 105 (6.6%) | 115 (7.5%) | | | 76 (6.3%) | 87 (8.0%) | | |
| 3 | 394 (25%) | 350 (23%) | | | 299 (25%) | 211 (19%) | | |
| 4 | 354 (22%) | 333 (22%) | | | 249 (21%) | 250 (23%) | | |
| 5 | 675 (42%) | 649 (43%) | | | 528 (44%) | 473 (44%) | | |
| 6 | 57 (3.6%) | 51 (3.3%) | | | 46 (3.8%) | 43 (4.0%) | | |
| **CAG** | 42.26 (2.05) | - | | | 42.22 (2.01) | - | | |
| **CAP score** | 283 (52) | - | | | 292 (53) | - | | |
| **TFC** | 13 (0) | 13 (0) | | | 13 (0) | 13 (0) | | |
| **UHDRS-TMS** | 1.03 (1.44) | 0.63 (1.17) | **<0.001** | -0.301 | 1.00 (1.42) | 0.62 (1.17) | **<0.001** | -0.297 |
| **MMSE** | 29.13 (1.24) | 29.20 (1.25) | 0.121 | 0.063 | 29.17 (1.20) | 29.36 (1.07) | **<0.001** | 0.160 |
| **SDMT** | 56 (11) | 57 (11) | 0.055 | 0.069 | 57 (11) | 58 (11) | 0.097 | 0.069 |
| **SCNT** | 78 (13) | 79 (13) | 0.055 | 0.069 | 79 (14) | 81 (13) | **0.045** | 0.084 |
| **SWRT** | 99 (16) | 100 (16) | **0.008** | 0.095 | 99 (17) | 101 (16) | **0.005** | 0.118 |
| **SCWT** | 48 (10) | 48 (10) | 0.407 | 0.030 | 49 (11) | 49 (10) | 0.523 | -0.027 |
| **Semantic Fluency** | 22.9 (5.4) | 23.4 (5.6) | **0.027** | 0.079 | 23.3 (5.4) | 23.8 (5.7) | 0.073 | 0.075 |
| **FAS** | 44 (13) | 43 (12) | 0.464 | -0.028 | 46 (13) | 45 (12) | 0.338 | -0.043 |
| **TMT-A** | 23 (9) | 23 (11) | 0.774 | -0.011 | 22 (8) | 22 (13) | 0.874 | 0.007 |
| **TMT-B** | 48 (23) | 47 (23) | 0.191 | -0.050 | 45 (20) | 45 (25) | 0.734 | -0.015 |

*Note:*Data are expressed as mean (SD) or n (%).
*Abbreviations*: HGECs, Huntingtin Gene Expansion Carriers; HC, Healthy Controls; ISCED, International Standard Classification of Education; CAP score, CAG age product; TFC, Total Functional Capacity; UHDRS-TMS, Unified Huntington's Disease Rating Scale – Total Motor Score; MMSE, Mini-mental State Examination; SDMT, Symbol Digit Modalities Test; SCNT, Stroop Color Naming Task; SWRT, Stroop Word Reading Task; SCWT, Stroop Color-Word Task; FAS, phonemic fluency; TMT, Trail Making Test.

**Supplementary Table 2.** (Continued)

| | V4 | | | | V5 | | | |
|---|---|---|---|---|---|---|---|---|
| | **HGECs** N = 858 | **HC** N = 810 | **p-value** | **Cohen's d** | **HGECs** N = 551 | **HC** N = 554 | **p-value** | **Cohen's d** |
| **Age** | 36 (8) | 39 (9) | **<0.001** | 0.297 | 37 (8) | 40 (9) | **<0.001** | -0.102 |
| **Sex** | | | 0.174 | | | | **0.021** | |
| female | 521 (61%) | 518 (64%) | | | 322 (58%) | 361 (65%) | | |
| male | 337 (39%) | 292 (36%) | | | 229 (42%) | 193 (35%) | | |
| **ISCED** | | | **0.001** | | | | **0.004** | |
| 1 | 6 (0.7%) | 23 (2.8%) | | | 1 (0.2%) | 14 (2.5%) | | |
| 2 | 47 (5.5%) | 67 (8.3%) | | | 32 (5.8%) | 33 (6.0%) | | |
| 3 | 204 (24%) | 160 (20%) | | | 141 (26%) | 112 (20%) | | |
| 4 | 180 (21%) | 181 (22%) | | | 106 (19%) | 108 (20%) | | |
| 5 | 386 (45%) | 347 (43%) | | | 243 (44%) | 267 (48%) | | |
| 6 | 34 (4.0%) | 31 (3.8%) | | | 28 (5.1%) | 19 (3.4%) | | |
| **CAG** | 42.24 (2.00) | - | | | 42.10 (1.95) | - | | |
| **CAP score** | 300 (53) | - | | | 305 (52) | - | | |
| **TFC** | 13 (0) | 13 (0) | | | 13(0) | 13(0) | | |
| **UHDRS-TMS** | 0.99 (1.45) | 0.60 (1.19) | **<0.001** | -0.296 | 1.09 (1.46) | 0.65 (1.21) | **<0.001** | 0.261 |
| **MMSE** | 29.32 (1.06) | 29.38 (1.00) | 0.362 | 0.051 | 29.39 (1.05) | 29.39 (1.03) | 0.967 | -0.100 |
| **SDMT** | 58 (11) | 59 (12) | 0.060 | 0.092 | 58 (11) | 60 (12) | **0.013** | -0.048 |
| **SCNT** | 80 (13) | 81 (13) | 0.057 | 0.093 | 80 (14) | 82 (13) | **0.046** | -0.043 |
| **SWRT** | 100 (17) | 102 (16) | **0.016** | 0.118 | 100 (18) | 102 (16) | **0.037** | -0.075 |
| **SCWT** | 50 (10) | 50 (10) | 0.911 | 0.006 | 50 (10) | 50 (10) | 0.236 | 0.046 |
| **Semantic Fluency** | 23.5 (5.5) | 23.8 (5.8) | 0.262 | 0.055 | 23.6 (5.4) | 24.2 (5.6) | 0.120 | -0.044 |
| **FAS** | 47 (13) | 47 (13) | 0.519 | -0.034 | 48 (12) | 47 (12) | 0.349 | -0.031 |
| **TMT-A** | 21 (7) | 21 (8) | 0.763 | 0.016 | 21 (7) | 21 (8) | 0.731 | 0.041 |
| **TMT-B** | 43 (19) | 43 (18) | 0.842 | -0.011 | 42 (17) | 42 (21) | 0.839 | 0.026 |

*Note:* Data are expressed as mean (SD) or n (%).
*Abbreviations*: HGECs, Huntingtin Gene Expansion Carriers; HC, Healthy Controls; ISCED, International Standard Classification of Education; CAP score, CAG age product; TFC, Total Functional Capacity; UHDRS-TMS, Unified Huntington's Disease Rating Scale – Total Motor Score; MMSE, Mini-mental State Examination; SDMT, Symbol Digit Modalities Test; SCNT, Stroop Color Naming Task; SWRT, Stroop Word Reading Task; SCWT, Stroop Color-Word Task; FAS, phonemic fluency; TMT, Trail Making Test.

**Supplementary Table 3. LME model coefficients**

| | SDMT | | | FAS | | | Semantic Fluency | | | TMT-A | | |
|---|---|---|---|---|---|---|---|---|---|---|---|---|
| | *Estimates* | *CI* | *p (FDR corrected)* | *Estimates* | *CI* | *p (FDR corrected)* | *Estimates* | *CI* | *p (FDR corrected)* | *Estimates* | *CI* | *p (FDR corrected)* |
| Age | -0.19 | -0.22 – -0.16 | **<0.001** | 0.11 | 0.07 – 0.15 | **<0.001** | 0.01 | -0.01 – 0.02 | 0.456 | 0.09 | 0.05 – 0.12 | **<0.001** |
| ISCED | 0.00 | -0.00 – 0.00 | 0.758 | -0.00 | -0.00 – 0.00 | 0.368 | -0.00 | -0.00 – 0.00 | 0.696 | 0.00 | -0.00 – 0.00 | 0.233 |
| Sex (male) | -2.70 | -3.24 – -2.16 | **<0.001** | -0.98 | -1.66 – -0.31 | **0.008** | -0.45 | -0.73 – -0.18 | **0.002** | -0.86 | -1.44 – -0.28 | **0.008** |
| Year 1 | 2.29 | 1.93 – 2.66 | **<0.001** | 1.63 | 1.19 – 2.08 | **<0.001** | 0.58 | 0.36 – 0.81 | **<0.001** | -1.47 | -1.93 – -1.02 | **<0.001** |
| Year 2 | 3.15 | 2.73 – 3.58 | **<0.001** | 3.32 | 2.81 – 3.83 | **<0.001** | 0.84 | 0.58 – 1.11 | **<0.001** | -2.25 | -2.77 – -1.72 | **<0.001** |
| Year 3 | 4.47 | 3.99 – 4.96 | **<0.001** | 4.35 | 3.77 – 4.93 | **<0.001** | 1.02 | 0.72 – 1.32 | **<0.001** | -3.18 | -3.77 – -2.58 | **<0.001** |
| Year 4 | 4.60 | 4.03 – 5.17 | **<0.001** | 4.68 | 3.99 – 5.36 | **<0.001** | 1.23 | 0.88 – 1.58 | **<0.001** | -3.52 | -4.22 – -2.82 | **<0.001** |
| Group (HGECs) | -0.70 | -1.26 – -0.14 | **0.025** | -0.15 | -0.85 – 0.54 | 0.705 | -0.25 | -0.55 – 0.04 | 0.142 | 0.53 | -0.10 – 1.15 | 0.145 |
| Year 1 x Group | -0.13 | -0.64 – 0.38 | 0.666 | 0.42 | -0.19 – 1.03 | 0.244 | -0.15 | -0.47 – 0.17 | 0.432 | -0.28 | -0.91 – 0.36 | 0.457 |
| Year 2 x Group | -0.43 | -1.01 – 0.15 | 0.206 | 0.05 | -0.64 – 0.75 | 0.884 | -0.13 | -0.49 – 0.23 | 0.549 | -0.27 | -0.99 – 0.44 | 0.522 |
| Year 3 x Group | -1.33 | -1.99 – -0.67 | **<0.001** | 0.12 | -0.67 – 0.91 | 0.78 | -0.22 | -0.63 – 0.19 | 0.362 | 0.14 | -0.67 – 0.95 | 0.76 |
| Year 4 x Group | -1.25 | -2.03 – -0.47 | **0.003** | -0.18 | -1.11 – 0.74 | 0.732 | -0.37 | -0.85 – 0.12 | 0.196 | 0.30 | -0.66 – 1.26 | 0.596 |

*Note:* Estimated coefficients with 95% confidence intervals (CI) and FDR-adjusted p-values.
*Abbreviations*: HGECs, Huntingtin Gene Expansion Carriers; ISCED, International Standard Classification of Education; SDMT, Symbol Digit Modalities Test; FAS, phonemic fluency; TMT, Trail Making Test.

**Supplementary Table 3.** (continued)

| | TMT-B | | | SCNT | | | SWRT | | | SCWT | | |
|---|---|---|---|---|---|---|---|---|---|---|---|---|
| | *Estimates* | *CI* | *p (FDR corrected)* | *Estimates* | *CI* | *p (FDR corrected)* | *Estimates* | *CI* | *p (FDR corrected)* | *Estimates* | *CI* | *p (FDR corrected)* |
| Age | 0.21 | 0.14 – 0.28 | **<0.001** | -0.10 | -0.14 – -0.06 | **<0.001** | -0.04 | -0.08 – 0.01 | 0.196 | -0.17 | -0.20 – -0.14 | **<0.001** |
| ISCED | 0.00 | -0.00 – 0.00 | 0.145 | -0.00 | -0.00 – 0.00 | 0.292 | -0.00 | -0.00 – -0.00 | **0.025** | -0.00 | -0.00 – 0.00 | 0.183 |
| Sex (male) | -0.15 | -1.40 – 1.11 | 0.829 | -0.87 | -1.55 – -0.19 | **0.021** | -0.25 | -1.08 – 0.57 | 0.6 | -0.29 | -0.81 – 0.23 | 0.343 |
| Year 1 | -3.92 | -4.98 – -2.86 | **<0.001** | 2.02 | 1.55 – 2.50 | **<0.001** | 1.07 | 0.49 – 1.66 | **<0.001** | 2.28 | 1.91 – 2.64 | **<0.001** |
| Year 2 | -5.31 | -6.52 – -4.09 | **<0.001** | 2.81 | 2.26 – 3.36 | **<0.001** | 1.81 | 1.14 – 2.49 | **<0.001** | 2.93 | 2.51 – 3.35 | **<0.001** |
| Year 3 | -7.98 | -9.36 – -6.60 | **<0.001** | 3.38 | 2.76 – 4.00 | **<0.001** | 2.50 | 1.73 – 3.26 | **<0.001** | 3.76 | 3.28 – 4.24 | **<0.001** |
| Year 4 | -7.86 | -9.48 – -6.23 | **<0.001** | 3.91 | 3.18 – 4.64 | **<0.001** | 2.61 | 1.71 – 3.51 | **<0.001** | 4.35 | 3.79 – 4.92 | **<0.001** |
| Group (HGECs) | 0.77 | -0.59 – 2.13 | 0.341 | -0.67 | -1.38 – 0.03 | 0.098 | -1.24 | -2.11 – -0.38 | **0.008** | 0.32 | -0.22 – 0.86 | 0.318 |
| Year 1 x Group | 0.50 | -0.97 – 1.97 | 0.566 | -0.40 | -1.06 – 0.26 | 0.306 | -0.50 | -1.31 – 0.32 | 0.306 | -0.90 | -1.41 – -0.40 | **<0.001** |
| Year 2 x Group | 0.65 | -1.02 – 2.31 | 0.515 | -0.88 | -1.62 – -0.13 | **0.035** | -1.12 | -2.04 – -0.20 | **0.028** | -0.41 | -0.98 – 0.17 | 0.228 |
| Year 3 x Group | 1.71 | -0.17 – 3.59 | 0.117 | -1.23 | -2.07 – -0.38 | **0.008** | -1.51 | -2.56 – -0.47 | **0.008** | -1.32 | -1.98 – -0.67 | **<0.001** |
| Year 4 x Group | 1.17 | -1.06 – 3.40 | 0.368 | -1.17 | -2.17 – -0.17 | **0.036** | -1.54 | -2.78 – -0.31 | **0.024** | -1.53 | -2.30 – -0.76 | **<0.001** |

*Note*: Estimated coefficients with 95% confidence intervals (CI) and FDR-adjusted p-values.
*Abbreviations*: HGECs, Huntingtin Gene Expansion Carriers: ISCED, International Standard Classification of Education; SCNT, Stroop Color Naming Task; SWRT, Stroop Word Reading Task; SCWT, Stroop Color-Word Task; TMT, Trail Making Test.

**Supplementary Table 4. Comparison of original and MICE-imputed linear mixed-effects model estimates for cognitive outcomes**

| Test | Interaction | β Original | β MICE | p Original | p MICE |
|---|---|---|---|---|---|
| **SDMT** | Year 1 x Group | -0.132 | -0.168 | 0.614 | 0.525 |
| | Year 2 x Group | -0.430 | -0.417 | 0.146 | 0.162 |
| | Year 3 x Group | -1.328 | -1.336 | 0.00008 | 0.00008 |
| | Year 4 x Group | -1.252 | -1.246 | 0.0016 | 0.0019 |
| **SCNT** | Year 1 x Group | -0.399 | -0.385 | 0.236 | 0.258 |
| | Year 2 x Group | -0.877 | -0.849 | 0.021 | 0.028 |
| | Year 3 x Group | -1.226 | -1.224 | 0.0046 | 0.0051 |
| | Year 4 x Group | -1.167 | -1.198 | 0.022 | 0.020 |
| **SWRT** | Year 1 x Group | -0.496 | -0.449 | 0.232 | 0.281 |
| | Year 2 x Group | -1.120 | -1.099 | 0.017 | 0.020 |
| | Year 3 x Group | -1.512 | -1.511 | 0.0045 | 0.0047 |
| | Year 4 x Group | -1.545 | -1.542 | 0.014 | 0.015 |
| **SCWT** | Year 1 x Group | -0.904 | -0.882 | 0.00047 | 0.00124 |
| | Year 2 x Group | -0.407 | -0.492 | 0.164 | 0.123 |
| | Year 3 x Group | -1.325 | -1.467 | 0.00007 | 0.00006 |
| | Year 4 x Group | -1.531 | -1.619 | 0.00010 | 0.00016 |
| **TMT-A** | Year 1 x Group | -0.277 | -0.192 | 0.391 | 0.602 |
| | Year 2 x Group | -0.271 | -0.184 | 0.457 | 0.680 |
| | Year 3 x Group | 0.142 | -0.055 | 0.731 | 0.904 |
| | Year 4 x Group | 0.300 | 0.324 | 0.539 | 0.531 |
| **TMT-B** | Year 1 x Group | 0.499 | 0.604 | 0.506 | 0.464 |
| | Year 2 x Group | 0.646 | 0.525 | 0.446 | 0.569 |
| | Year 3 x Group | 1.710 | 1.323 | 0.075 | 0.219 |
| | Year 4 x Group | 1.171 | 1.118 | 0.303 | 0.355 |
| **Semantic Fluency** | Year 1 x Group | -0.148 | -0.145 | 0.361 | 0.374 |
| | Year 2 x Group | -0.128 | -0.158 | 0.486 | 0.391 |
| | Year 3 x Group | -0.219 | -0.229 | 0.292 | 0.273 |
| | Year 4 x Group | -0.367 | -0.377 | 0.136 | 0.128 |
| **FAS** | Year 1 x Group | 0.419 | 0.354 | 0.180 | 0.377 |
| | Year 2 x Group | 0.052 | 0.019 | 0.884 | 0.962 |
| | Year 3 x Group | 0.120 | -0.011 | 0.765 | 0.980 |
| | Year 4 x Group | -0.184 | 0.125 | 0.697 | 0.840 |

*Abbreviations*: SDMT, Symbol Digit Modalities Test; SCNT, Stroop Color Naming Task; SWRT, Stroop Word Reading Task; SCWT, Stroop Color-Word Task; TMT, Trail Making Test; FAS, phonemic fluency.

**Supplementary Table 5. Segmented regression analysis**

| | SDMT | | | SCNT | | | SWRT | | | SCWT | | |
|---|---|---|---|---|---|---|---|---|---|---|---|---|
| *Predictors* | *Estimates* | *CI* | *p* | *Estimates* | *CI* | *p* | *Estimates* | *CI* | *p* | *Estimates* | *CI* | *p* |
| **Intercept** | 60.69 | 58.01 – 63.36 | **<0.001** | 82.13 | 78.11 – 86.15 | **<0.001** | 99.31 | 93.15 – 105.46 | **<0.001** | 48.93 | 45.44 – 52.41 | **<0.001** |
| **Year 1** | 4.30 | 0.75 – 7.85 | **0.017** | 4.64 | 0.16 – 9.12 | **0.042** | 4.67 | -0.95 – 10.29 | 0.104 | 2.48 | -1.03 – 5.98 | 0.166 |
| **Year 2** | 7.81 | 3.81 – 11.82 | **<0.001** | 5.46 | 0.41 – 10.50 | **0.034** | 5.36 | -0.96 – 11.67 | 0.097 | 4.54 | 0.57 – 8.51 | **0.025** |
| **Year 3** | 7.02 | 2.32 – 11.72 | **0.003** | 5.08 | -0.82 – 10.97 | 0.091 | 5.86 | -1.51 – 13.24 | 0.119 | 4.62 | -0.01 – 9.25 | 0.050 |
| **Year 4** | 9.00 | 3.20 – 14.80 | **0.002** | 5.29 | -1.98 – 12.56 | 0.154 | 6.17 | -2.93 – 15.27 | 0.184 | 1.26 | -4.40 – 6.91 | 0.663 |
| **CAP (before breakpoint)** | -0.02 | -0.03 – -0.01 | **<0.001** | -0.02 | -0.03 – 0.00 | 0.057 | -0.00 | -0.03 – 0.03 | 0.994 | -0.00 | -0.02 – 0.01 | 0.563 |
| **CAP (after breakpoint)** | -0.05 | -0.07 – -0.03 | **<0.001** | -0.04 | -0.06 – -0.02 | **0.001** | -0.04 | -0.07 – -0.01 | **0.011** | -0.03 | -0.05 – -0.01 | **0.001** |
| **Year 1 x CAP** | -0.00 | -0.02 – 0.01 | 0.484 | -0.00 | -0.02 – 0.01 | 0.783 | -0.01 | -0.03 – 0.01 | 0.389 | -0.00 | -0.02 – 0.01 | 0.601 |
| **Year 2 x CAP** | -0.01 | -0.02 – 0.00 | 0.125 | -0.00 | -0.02 – 0.02 | 0.909 | -0.01 | -0.03 – 0.01 | 0.377 | 0.01 | -0.01 – 0.02 | 0.223 |
| **Year 3 x CAP** | -0.00 | -0.02 – 0.01 | 0.598 | -0.00 | -0.02 – 0.02 | 0.818 | -0.01 | -0.03 – 0.02 | 0.498 | -0.00 | -0.02 – 0.01 | 0.671 |
| **Year 4 x CAP** | -0.01 | -0.03 – 0.01 | 0.279 | -0.00 | -0.03 – 0.02 | 0.926 | -0.01 | -0.04 – 0.02 | 0.529 | 0.01 | -0.01 – 0.03 | 0.378 |

*Abbreviations*: CAP score, CAG age product; SDMT, Symbol Digit Modalities Test; SCNT, Stroop Color Naming Task; SWRT, Stroop Word Reading Task; SCWT, Stroop Color-Word Task.

**Supplementary Table 6. Estimated breakpoints**

| Test | Estimated Breakpoint | 95% CI lower | 95% CI upper |
|---|---|---|---|
| SWRT | 249.56 | 210.27 | 288.85 |
| SCWT | 258.50 | 228.88 | 288.13 |
| SCNT | 268.84 | 237.41 | 300.27 |
| SDMT | 291.90 | 272.734 | 311.07 |

*Abbreviations*: SWRT, Stroop Word Reading Task; SCWT, Stroop Color-Word Task; SCNT, Stroop Color Naming Task; SDMT, Symbol Digit Modalities Test.

**Supplementary Table 7. LME analysis stratified by CAP score quartiles**

| | SDMT | | | SCNT | | | SWRT | | | SCWT | | |
|---|---|---|---|---|---|---|---|---|---|---|---|---|
| *Predictors* | *Estimates* | *CI* | *p* | *Estimates* | *CI* | *p* | *Estimates* | *CI* | *p* | *Estimates* | *CI* | *p* |
| **Intercept** | 59.46 | 57.88 – 61.04 | **<0.001** | 79.80 | 77.82 – 81.79 | **<0.001** | 98.66 | 96.16 – 101.16 | **<0.001** | 50.20 | 48.63 – 51.76 | **<0.001** |
| **Age** | -0.09 | -0.14 – -0.04 | **<0.001** | -0.02 | -0.08 – 0.05 | 0.609 | 0.04 | -0.04 – 0.12 | 0.325 | -0.08 | -0.13 – -0.03 | **0.003** |
| **ISCED** | 0.00 | -0.00 – 0.00 | 0.065 | -0.00 | -0.00 – 0.00 | 0.674 | -0.00 | -0.00 – 0.00 | 0.231 | 0.00 | -0.00 – 0.00 | 0.931 |
| **Sex (male)** | -2.14 | -2.89 – -1.38 | **<0.001** | -0.91 | -1.85 – 0.04 | 0.059 | -0.26 | -1.45 – 0.93 | 0.664 | -0.21 | -0.96 – 0.53 | 0.578 |
| **Year 1** | 2.55 | 1.85 – 3.25 | **<0.001** | 2.40 | 1.48 – 3.32 | **<0.001** | 1.12 | 0.00 – 2.24 | **0.050** | 1.86 | 1.16 – 2.56 | **<0.001** |
| **Year 2** | 3.63 | 2.83 – 4.43 | **<0.001** | 2.73 | 1.68 – 3.78 | **<0.001** | 0.96 | -0.32 – 2.23 | 0.140 | 2.96 | 2.15 – 3.76 | **<0.001** |
| **Year 3** | 3.75 | 2.84 – 4.67 | **<0.001** | 2.88 | 1.69 – 4.08 | **<0.001** | 2.34 | 0.89 – 3.79 | **0.002** | 3.49 | 2.58 – 4.41 | **<0.001** |
| **Year 4** | 4.45 | 3.36 – 5.54 | **<0.001** | 3.51 | 2.08 – 4.93 | **<0.001** | 1.83 | 0.11 – 3.56 | **0.037** | 3.17 | 2.09 – 4.25 | **<0.001** |
| **Q2** | -0.91 | -2.04 – 0.22 | 0.113 | -1.08 | -2.50 – 0.34 | 0.135 | -1.10 | -2.88 – 0.69 | 0.228 | -0.10 | -1.22 – 1.01 | 0.855 |
| **Q3** | -2.02 | -3.21 – -0.83 | **0.001** | -3.63 | -5.13 – -2.12 | **<0.001** | -3.42 | -5.31 – -1.53 | **<0.001** | -1.70 | -2.88 – -0.52 | **0.005** |
| **Q4** | -3.57 | -4.83 – -2.32 | **<0.001** | -4.16 | -5.74 – -2.58 | **<0.001** | -3.75 | -5.74 – -1.76 | **<0.001** | -1.85 | -3.10 – -0.61 | **0.004** |
| **Year 1 x Q2** | -0.03 | -1.03 – 0.96 | 0.949 | -0.87 | -2.17 – 0.43 | 0.191 | -0.09 | -1.67 – 1.49 | 0.911 | -0.48 | -1.47 – 0.51 | 0.346 |
| **Year 2 x Q2** | -0.92 | -2.03 – 0.19 | 0.106 | -0.67 | -2.12 – 0.78 | 0.366 | 0.73 | -1.04 – 2.49 | 0.419 | -0.34 | -1.45 – 0.77 | 0.552 |
| **Year 3 x Q2** | 0.17 | -1.09 – 1.43 | 0.796 | -0.21 | -1.86 – 1.44 | 0.803 | -0.69 | -2.70 – 1.31 | 0.496 | -1.14 | -2.40 – 0.13 | 0.078 |
| **Year 4 x Q2** | -0.50 | -1.99 – 0.99 | 0.513 | 0.69 | -1.26 – 2.64 | 0.486 | 1.00 | -1.36 – 3.36 | 0.405 | -0.23 | -1.70 – 1.25 | 0.765 |
| **Year 1 x Q3** | -0.98 | -1.97 – 0.02 | 0.054 | -1.12 | -2.42 – 0.19 | 0.093 | -1.07 | -2.65 – 0.51 | 0.184 | -0.28 | -1.27 – 0.72 | 0.586 |
| **Year 2 x Q3** | -1.32 | -2.44 – -0.20 | **0.021** | -1.29 | -2.76 – 0.17 | 0.084 | -0.98 | -2.75 – 0.80 | 0.281 | -0.28 | -1.41 – 0.84 | 0.624 |
| **Year 3 x Q3** | -2.05 | -3.32 – -0.79 | **0.001** | -1.74 | -3.39 – -0.09 | **0.039** | -3.30 | -5.30 – -1.29 | **0.001** | -1.80 | -3.07 – -0.53 | **0.005** |
| **Year 4 x Q3** | -2.65 | -4.14 – -1.16 | **<0.001** | -2.36 | -4.30 – -0.42 | **0.017** | -2.73 | -5.08 – -0.37 | **0.023** | -0.44 | -1.92 – 1.04 | 0.557 |
| **Year 1 x Q4** | -1.01 | -2.01 – -0.01 | **0.049** | -1.53 | -2.85 – -0.22 | **0.022** | -1.44 | -3.03 – 0.15 | 0.076 | -1.66 | -2.66 – -0.67 | **0.001** |
| **Year 2 x Q4** | -2.26 | -3.39 – -1.13 | **<0.001** | -1.97 | -3.45 – -0.49 | **0.009** | -1.61 | -3.40 – 0.17 | 0.077 | -2.01 | -3.14 – -0.88 | **0.001** |
| **Year 3 x Q4** | -1.90 | -3.21 – -0.60 | **0.004** | -2.11 | -3.83 – -0.40 | **0.016** | -2.56 | -4.64 – -0.48 | **0.016** | -2.61 | -3.92 – -1.29 | **<0.001** |
| **Year 4 x Q4** | -3.34 | -4.97 – -1.71 | **<0.001** | -3.33 | -5.46 – -1.19 | **0.002** | -3.26 | -5.84 – -0.68 | **0.013** | -2.83 | -4.47 – -1.19 | **0.001** |

*Abbreviations:* ISCED, International Standard Classification of Education; SDMT, Symbol Digit Modalities Test; SCNT, Stroop Color Naming Task; SWRT, Stroop Word Reading Task; SCWT, Stroop Color-Word Task; Q1-Q4, Quartiles 1-4.

**Supplementary Table 8. Internal validation of the normative PE-TraC model in the held-out HC test set**

| Test | N pairs | N subjects | Mean z | SD z | Coverage ±1.96 SD (%) |
|---|---|---|---|---|---|
| **SCNT** | 1,662 | 348 | -0.085 | 1.21 | 91.2 |
| **SCWT** | 1,513 | 331 | -0.140 | 1.12 | 92.7 |
| **SDMT** | 1,658 | 348 | -0.085 | 1.28 | 88.2 |
| **SWRT** | 1,665 | 348 | -0.051 | 1.22 | 89.6 |

*Note:*Mean z indicates centering of standardized residuals around zero; SD z reflects dispersion relative to the expected unit variance; coverage corresponds to the proportion of observations within ±1.96 SD in the held-out HC test set. *Abbreviations*: SDMT, Symbol Digit Modalities Test; SCNT, Stroop Color Naming Task; SWRT, Stroop Word Reading Task; SCWT, Stroop Color-Word Task; HC, Healthy Controls.

**Supplementary Table 9. Internal validation of the normative PE-TraC model by visit lag in the held-out HC test set**

| Test | Lag | N pairs | Mean z | SD z | Coverage ±1.96 SD (%) |
|---|---|---|---|---|---|
| **SCNT** | 1 | 772 | −0.043 | 1.26 | 91.2 |
| **SCNT** | 2 | 489 | −0.111 | 1.18 | 90.8 |
| **SCNT** | 3 | 277 | −0.122 | 1.15 | 91.3 |
| **SCNT** | 4 | 124 | −0.157 | 1.05 | 92.7 |
| **SCWT** | 1 | 709 | −0.104 | 1.18 | 91.9 |
| **SCWT** | 2 | 445 | −0.183 | 1.05 | 93.5 |
| **SCWT** | 3 | 248 | −0.153 | 1.08 | 93.1 |
| **SCWT** | 4 | 111 | −0.162 | 1.10 | 93.7 |
| **SDMT** | 1 | 770 | −0.033 | 1.29 | 88.3 |
| **SDMT** | 2 | 488 | −0.127 | 1.26 | 89.3 |
| **SDMT** | 3 | 277 | −0.132 | 1.22 | 87.7 |
| **SDMT** | 4 | 123 | −0.136 | 1.43 | 84.6 |
| **SWRT** | 1 | 773 | −0.027 | 1.27 | 89.5 |
| **SWRT** | 2 | 490 | −0.038 | 1.19 | 89.2 |
| **SWRT** | 3 | 278 | −0.090 | 1.15 | 90.3 |
| **SWRT** | 4 | 124 | −0.161 | 1.19 | 90.3 |

*Note:* Validation metrics are shown separately by visit lag, defined as the number of visit intervals between the reference and comparison assessments. *Abbreviations*: HC, Healthy Controls.
